# The Utility of Visual and Olfactory Maize Leaf Cues in Host Finding by Adult *Spodoptera frugiperda* (Lepidoptera: Noctuidae)

**DOI:** 10.3390/plants13233300

**Published:** 2024-11-25

**Authors:** Jie Liu, Mariam Tallat, Gensong Wang, Guoping Li, Haowen Zhang, Xujin Wu, Hongbo Qiao, Xincheng Zhao, Hongqiang Feng

**Affiliations:** 1 Henan Key Laboratory of Agricultural Pest Monitoring and Control, IPM Key Laboratory in Southern Part of North China for Ministry of Agriculture, International Joint Research Laboratory for Crop Protection of Henan, No. 0 Entomological Radar Field Scientific Observation and Research Station of Henan Province, Institute of Plant Protection, Henan Academy of Agricultural Sciences, Zhengzhou 450002, Chinamariamtallat.4412@gmail.com (M.T.); hn2002wgs@163.com (G.W.); liguoping1976@163.com (G.L.); 2 College of Plant Protection, Henan Agricultural University, Zhengzhou 450046, China; xincheng@henau.edu.cn; 3 State Key Laboratory for Biology of Plant Diseases and Insect Pests, Institute of Plant Protection, Chinese Academy of Agricultural Sciences, Beijing 100193, China; marszhang_0@163.com; 4 Institute of Quality and Safety for Agro-Products, Henan Academy of Agricultural Sciences, Zhengzhou 450002, China; xujinwu2005@126.com; 5 College of Information and Management Science, Henan Agricultural University, Zhengzhou 450046, China; qiaohb@henau.edu.cn

**Keywords:** *Spodoptera frugiperda*, host plant location, maize, visual cue, olfactory cue, volatile organic compounds

## Abstract

The fall armyworm, *Spodoptera frugiperda* (Lepidoptera: Noctuidae) (FAW), is an invasive and destructive polyphagous pest that poses a significant threat to global agricultural production. The FAW mainly damages maize, with a particular preference for V3–V5 (third to fifth leaf collar) plant stages in northern China. How the FAW moth precisely locates maize plants in the V3–V5 stage at night remains unclear. The aims of this study were to evaluate the visual and olfactory cues used by the FAW to identify its host plant, maize, in order to select attractants with better trapping efficacy. Hyperspectral analysis of maize plants at different growth stages using the ASD Fieldspec 4 spectrometer was performed using mimics (moths or maize leaves sealed with transparent plastic sheets) and black cloth-covered plants for single visual and single olfactory attraction experiments. Gas chromatography–mass spectrometry (GC-MS) was used to analyze volatiles emitted from V3–V5 stage maize leaves. Volatile organic chemicals (VOCs) were screened using electroantennography (EAG) and Y-tube. Attractor efficacy was validated using mimics + VOCs. Results showed very little variance in the spectral reflectance curve of the maize at different growth stages. Fifteen VOCs were identified in the V3–V5 stage leaves of three different maize varieties, of which *cis*-3-hexenyl acetate and myrcene were found in relatively high concentrations in these maize varieties. The frequency of visits attracted by single visual stimuli was significantly lower than that attracted by single olfactory or olfactory + visual cues. The attractiveness of foliar *cis*-3-hexenyl acetate increased as its concentration decreased. The combination of mimics + *cis*-3-hexenyl acetate (1 ng/μL) increased host detection efficiency and stimulated mating behavior. These results indicate that the nocturnal insect FAW primarily uses olfactory cues for host identification, with visual cues serving as a complementary modality. The synergistic effect of olfactory and visual cues increases the efficiency of host recognition. We found that *cis*-3-hexenol acetate at a concentration from maize leaves is a reliable olfactory signal for the FAW. When using host plant VOCs as attractants to control adult FAWs, the role of visual cues must be considered.

## 1. Introduction

Plants and insects have coexisted for the past 350 million years, during which time various forms of interactions between them have evolved [1]. Host plants provide a variety of benefits to herbivorous insects, including mating sites [2,3,4], food sources for offspring development [4,5,6], and oviposition sites [7,8]. Food and reproduction are the driving factors for herbivorous insects in their search for suitable habitats. The selection of a host plant by an insect is a complex process involving a number of factors that ultimately lead to acceptance and feeding on the chosen host [9,10]. The evolution of volatiles to attract insects that protect the plant or to repel species that damage it (since these are the signals that insects use as olfactory cues) is a co-evolutionary relationship that has resulted in a sophisticated interplay between insect sensory systems and plant traits. Insects have evolved the ability to detect and respond to various visual and chemical plant cues. Visual cues (color and shape) [11,12,13,14] and olfactory cues (airborne/volatile chemical signals) play important roles in host location and acceptance processes [12,15,16,17].

Particularly for Lepidopteran insects, adult oviposition site selection can significantly affect offspring survival, competition, growth rate, and defense against predators and parasites [18,19]. Because larvae typically remain on their natal host plant from hatching through the later larval stages [20,21,22,23], their performance depends on both the nutritional value of the plant and the potential protection against predators and parasites that the larvae can obtain from the plant [24]. The trichromatic vision of insects allows them to perceive primarily ultraviolet, blue, and green light [25]. Plant leaf volatiles can serve as olfactory cues to attract Lepidopteran insects [26,27]. Studies have shown that visual and chemical cues play a particularly important role in the host location and acceptance processes of the cabbage moth, *Mamestra brassicae* [28]. Research on *M. brassicae* provides valuable insights into the complex interactions between a generalist herbivore and its host plants and offers novel strategies for pest control through the manipulation of chemical signals. *S. frugiperda* is more sensitive to blue light than to other types of light [29]. Maize leaf VOCs mediate the host and oviposition preferences of *S. frugiperda* [8]. Plant attractiveness to insects depends on key compounds and their concentrations in the volatile mixture [30,31,32]. The suitability of the Y-tube olfactometer and mimics for studying the behavioral responses of lepidopteran insects has been reported in several studies [33,34,35,36].

The fall armyworm *Spodoptera frugiperda* (FAW) is a polyphagous pest native to the tropical and subtropical regions of the Americas that feeds on 353 plant species from 76 families. The FAW causes damage to crops primarily through direct consumption of plant leaves and tunneling by its larvae [37,38]. Global populations of fall armyworm can be divided into two phenotypically similar but genetically distinct subpopulations or strains: the rice strain (RS) and the maize strain (CS) [38]. In January 2019, the FAW invaded China and has since been feeding primarily on maize throughout the country [39]. It has rapidly spread to most regions of China in a short period of time (by October 2019, it had spread to 26 provinces in China), feeding not only on maize but also on other crops such as rice, sugarcane, sorghum, and millet [40,41,42]. The alarming spread can be attributed to the pest’s rapid migratory ability, high reproductive potential, polyphagy, and the difficulty of control [43,44]. Visual and olfactory cues can influence insect preferences in host plant selection. The FAW and some other moths in Noctuidae are primarily nocturnal, seeking oviposition hosts and habitats at night [8]. However, finding host plants in the dimly lit nocturnal environment is no easy task! To gain a comprehensive understanding of the host-finding mechanisms of the FAW, it is necessary to assess the response to a combination of olfactory and visual cues (bimodal cues). To date, there are no reports on the role of visual and olfactory leaf cues in adult host finding. In this study, we combine visual and chemical methods for the first time to describe how adult FAWs accomplish this. The objectives of this research are to elucidate the use of vision and olfaction in the host recognition process of adult FAWs, whether they can identify host plants using a single visual cue (leaf color), whether there are differences in visual signals between different maize cultivars and the same cultivar at different growth stages, and whether visual signals are consistent across cultivars at the same growth stage. In addition to vision, is olfaction a primary cue involved in host recognition? Are plant VOCs the main factor influencing recognition of their host plant, maize, and are VOCs from the small trumpet stage V3–V5 (third to fifth leaf collar) of maize more effective in improving host recognition or stimulating courtship behavior? Or, does the combined use of color and odor enhance recognition? The precise elucidation of the host-recognition mechanism and selection of ideal lures are prerequisites for formulating pest-management strategies. Understanding the weights of olfactory and visual signals that mediate the host recognition behavior of adult FAWs may help to develop new traps and improve the efficiency of ecological control of crop pests.

## 2. Results

### 2.1. Field Damage to Maize Plants by FAW Larvae

**Figure 1:**
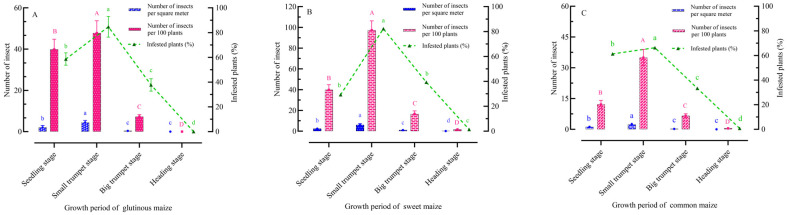
Field damage caused by FAW larvae (**A**) on glutinous maize, (**B**) on sweet maize, (**C**) on common maize. Histograms are expressed as mean ± SD. One-way ANOVA was used to determine differences in field damage caused by FAW larvae (*p* < 0.05). Post hoc test was used by LSD. Uppercase letters indicate significant differences in the number of insects per 100 plants. Blue lowercase letters indicate significant differences in the number of insects per square meter. Green lowercase letters indicate significant differences in infested plants (%).

The responses indicate that there are differences in the degree of larval damage at different growth stages for the same maize variety (Figure 1). For three different maize varieties, the most severe damage to maize plants occurred at the small trumpet stage. The number of insects per square meter, the number of insects per 100 plants, and the percentage of infested plants for the three different varieties at the small trumpet stage were as follows: glutinous maize (4.44, 47.74, 50.82%, respectively), sweet maize (5.04, 97.24, 98.74%, respectively), and common maize (4.01, 34.95, 39.80%, respectively).

### 2.2. Reflectance Spectra of Maize Leaf and Maize Leaf + Soil Mixed

**Figure 2:**
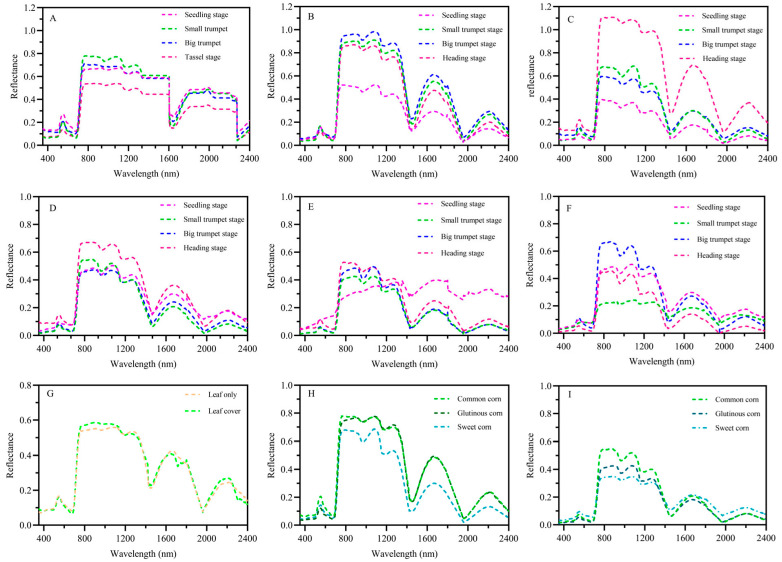
Spectral reflectance of maize leaves and combined leaf/soil. (**A**) Common maize leaves. (**B**) Glutinous maize leaves. (**C**) Sweet maize leaves. (**D**) Common maize combined leaf/soil. (**E**) Glutinous maize combined leaf/soil. (**F**) Sweet maize combined leaf/soil. (**G**) Leaf only and leaf cover (mimics). (**H**) Maize leaves at small trumpet stage. (**I**) Combined leaf/soil at small trumpet stage. A chi-squared test (L-R *X*^2^ degrees of freedom = 1) was used to detect significant differences between the leaf reflectance spectra of leaf only and leaf cover.

The first small peak of the spectrum (490~570 nm) and the spectral curves were almost the same for the same maize variety and for different maize varieties during the same growth period (Figure 2). The spectral reflectance of a single maize leaf was stronger than that of mixed leaf + soil samples (Figure 2H,I). The spectral curves and major peaks between the samples and the mimics were almost identical, with only slight differences in reflectance (Figure 2G). This suggests that there is no significant difference between the mimics and the samples and that there is little effect on the perception of color vision in adults (Pearson L-R *X*^2^ = 1.147A, df = 1, *p* > 0.05).

### 2.3. Single Visual Recognition by Adult FAWs During Host Search

**Figure 3:**
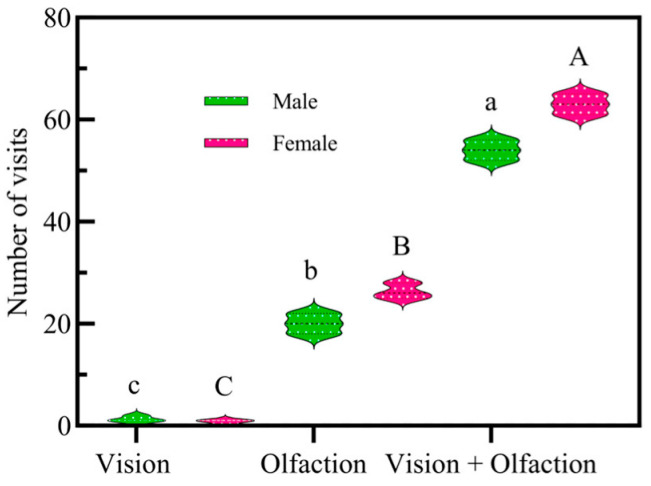
Comparison of visual or olfactory responses of adult FAWs to host plants. Different letters indicate significant differences in the number of visits under three different conditions by males (lowercase letters) and by females (uppercase letters).

The overall frequency of adult FAW visits to three different maize varieties during the process of a single visual identification of the host plant was relatively low (1~3 times) (Figure 3). The frequency of adult visits to the same maize variety at different growth stages showed no significant difference, nor did the frequency of visits to different varieties at the same growth stage show any significant difference. Differences in visual recognition of the host plant maize by male and female adults were not significant (*t* = 2.0, df = 4, *p* > 0.05). The combined effect of adult sex and maize growth stage had no significant effect on adult visits to sweet maize (F = 0.741, df = 3, *p* > 0.05). The combined effect of adult sex and maize growth stage had no significant effect on adult visits to glutinous maize (F = 0.121, df = 3, *p* > 0.05). The combined effect of adult sex and maize growth stage had no significant effect on adult visits to common maize (F = 2.389, df = 3, *p* > 0.05). The combined effect of adult sex and maize variety had no significant effect on adult visits to small trumpet stage maize (F = 1.75, df = 2, *p* > 0.05).

### 2.4. Single Olfactory Recognition by Adult FAWs During Host Search

Both male and female adults were more active, with visitation frequencies of 26 times (56.52%) for females and 20 times (43.48%) for males during the single odor recognition process of the host plant. The visitation frequency of female adults was significantly higher than that of males (F = 0.082, df = 4, *p* < 0.05) (Figure 3).

### 2.5. Visual and Olfactory Responses by Adult FAWs During Host Search

During the host recognition process, the integrated use of vision and olfaction resulted in the highest visit frequency, with the number of visits being 63 times (53.85%) for females and 54 times (46.15%) for males (Figure 3). The visit frequency for olfaction was much higher than for vision (F = 1.73, df = 4, *p* < 0.01), indicating that olfaction plays a dominant role. The visitation frequency of male adults with combined vision and olfaction was 29.83 times that of single vision, and that of females was 98.43 times. The visitation frequency of male adults with combined vision and olfaction was 2.7 times that of single olfaction, and that of females was 2.42 times.

### 2.6. Analysis of Maize Leaves’ VOCs

Based on the results of previous field surveys of damage caused by larvae that show a preference for feeding on maize at the V3–V5 stage, this study conducted a volatile analysis on maize of three different cultivars at the V3–V5 stage. The VOCs detected in this study can be categorized into four groups: green leaf VOCs, terpenoids, aliphatics (alcohols, aldehydes, esters, hydrocarbons), and aromatics. A total of fifteen VOCs were identified from maize leaves of three different species (Table 1). A total of eleven VOCs were detected in sweet maize, thirteen in common maize, and eleven in glutinous maize. Seven VOCs were common to three different cultivars. *cis*-3-hexenyl acetate (50.21, 66.37, 98.37, respectively) was a common and relatively high-concentration VOC found in sweet maize, regular maize and waxy maize. It was followed by myrcene, linalool, benzaldehyde, and ethylhexyl acetate. Perillen and octene were unique to sweet maize.

### 2.7. EAG Responses to Maize Leaf VOCs by Adult FAWs

**Figure 4:**
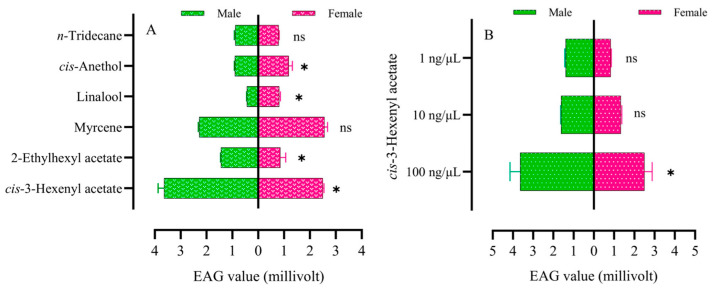
EAG responses of adult FAWs to host plant VOCs: (**A**) to different VOCs at a concentration of 100 μg/μL; (**B**) to different concentrations of *cis*-3-hexenyl acetate. Histograms are expressed as mean ± SD. Differential EAG values between female and male adult FAWs were analyzed by *t*-test. * indicates statistically significant differences (*p* < 0.05) in EAG values between males and females. ns indicates no significant differences (*p* > 0.05) in EAG values between males and females.

Adult EAG response peaks to six different types of VOCs varied (Figure 4A), with the values in descending order: *cis*-3-hexenyl acetate > myrcene > 2-ethylhexyl acetate > *cis*-anethol > *n*-tridecane > linalool. Male and female adult peak responses to the VOCs *n*-tridecane (F = 1.60, df = 4, *p* > 0.05) and myrcene (F = 0.308, df = 4, *p* < 0.01) showed no significant differences (Figure 4A). Male and female adult peak responses to 1 ng/µL *cis*-3-hexenyl acetate (F = 0.613, df = 4, *p* > 0.05) and 10 ng/µL *cis*-3-hexenyl acetate (F = 0.679, df = 4, *p* > 0.05) were not significantly different (Figure 4B). The EAG response peaks of male and female adults to VOC 100 ng/µL *cis*-3-hexenyl acetate were significantly different (F = 8.00, df = 4, *p* < 0.05) (Figure 4B). The EAG response peaks of adult FAWs to *cis*-3-hexenyl acetate at different concentrations showed a difference, with the response decreasing as the concentration decreased (Figure 4B).

### 2.8. Y-Tube Responses to Common Maize Leaf VOCs by Adult FAWs

**Figure 5:**
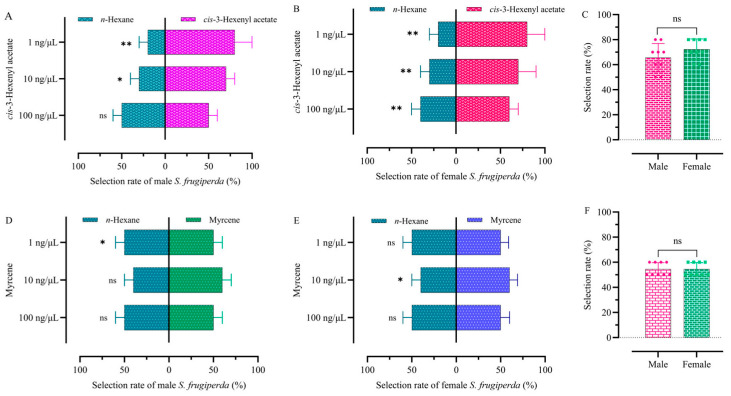
Selection rate of FAWs to maize leaf VOCs. (**A**) Males to *cis*-3-hexenyl acetate. (**B**) Females to *cis*-3-hexenyl acetate. (**C**) Comparison of selection rates to *cis*-3-hexenyl acetate. (**D**) Males to myrcene. (**E**) Females to myrcene. Histograms are expressed as mean ± SD. (**F**) Comparison of selection rates to myrcene. Selection rate between female and male adults was analyzed by *t*-test. * indicates statistically significant differences (*p* < 0.05). ** indicates statistically significant differences (*p* < 0.01). ns indicates no significant differences.

Adult sexes differed in their attraction to different VOCs and to different concentrations of the same VOC (Figure 5). Males showed no response to 100 ng/μL *cis*-3-hexenyl acetate, whereas female moths showed a strong behavioral attraction to it (Figure 5A,B). Myrcene at 1 ng/μL showed strong attraction for adult males, whereas myrcene at 10 ng/μL showed strong attraction for adult females (Figure 5D,E). There was no significant difference in the selectivity of adult males and females for different concentrations of *cis*-3-hexenyl acetate (F = 0.727, df = 39, *p* > 0.05) (Figure 5C). There was no significant difference in the selectivity of adult males and females for different concentrations of myrcene (F = 0.879, df = 31, *p* > 0.05) (Figure 5E).

### 2.9. Leaf Mimics + Different Concentration VOCs Attraction Experiment

**Figure 6:**
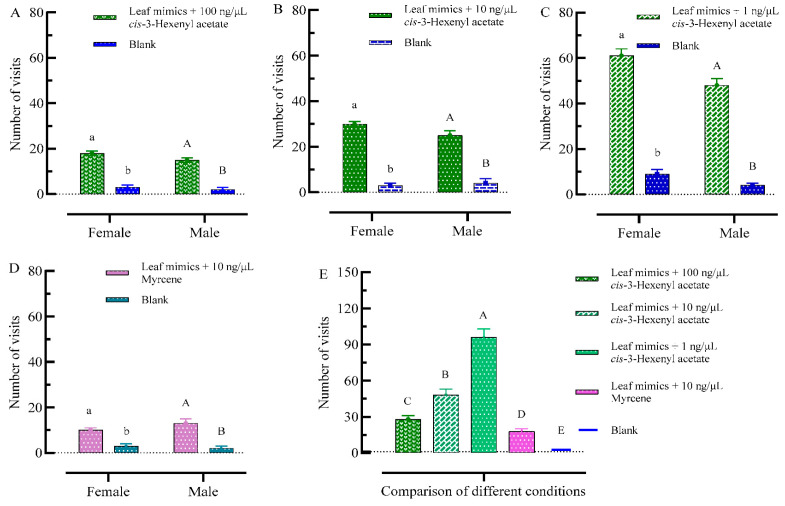
Comparison of numbers of adult visits to models + VOCs with single leaf mimics. (**A**) Comparison of numbers of adult visits to mimics + 100 ng/μL *cis*-3-hexenyl acetate with single model. (**B**) Comparison of numbers of adult visits to mimics + 10 ng/μL *cis*-3-hexenyl acetate with single model. (**C**) Comparison of numbers of adult visits to mimics + 1 ng/μL *cis*-3-hexenyl acetate with single model. (**D**) Comparison of numbers of adult visits to mimics + 10 ng/μL myrcene with single model. (**E**) Comparison of the total numbers of adult visits to five different conditions. Histograms are expressed as mean ± SD. Differences in the numbers of adult visits (*p* < 0.05) were determined by one-way ANOVA. Post hoc test is used with LSD. Lowercase letters indicate significant differences in female visits to mimics (Figure 6A–D). Uppercase letters indicate significant differences in male visits to mimics (Figure 6A–D). Uppercase letters indicate significant differences in adult visits to five different conditions (Figure 6E).

The results showed that both male and female adults visited the mimics plus VOCs more frequently than the control (Figure 6A–D). The total number of adult FAW visits to the 100 ng/μL *cis*-3-hexenyl acetate + leaf mimics was 33, for males and females 18 and 15, respectively, which was significantly greater than that to the blank (F = 22.216; df = 113, *p* < 0.01) (Figure 6A). The total number of adult FAW visits to the 10 ng/μL *cis*-3-hexenyl acetate + leaf mimics was 55, for males and females 25 and 30, respectively, which was significantly greater than that to the blank (F = 28.831; df = 185, *p* < 0.01) (Figure 6B). The total number of adult FAW visits to the 1 ng/μL *cis*-3-hexenyl acetate + leaf mimics was 109, for males and females 48 and 61, respectively, which was significantly greater than that to the blank (F = 29.203; df = 324, *p* < 0.01) (Figure 6C). The total number of adult FAW visits to the 10 ng/μL myrcene + leaf mimics was 23, for males and females 13 and 10, respectively, which was significantly greater than that to the blank (F = 23.757; df = 83, *p* < 0.01) (Figure 6D). The visit frequency of 1 ng/μL *cis*-3-hexenyl acetate + leaf mimics was significantly different from that of 100 ng/μL *cis*-3-hexenyl acetate + leaf mimics and 10 ng/μL *cis*-3-hexenyl acetate + leaf mimics (F = 124.909; df = 2, *p* < 0.01) (Figure 6E). The visit frequency of adults to *cis*-3-hexenyl acetate increased with decreasing concentration, and the difference in visit frequency of male and female adults to the same concentration of 10 ng/μL of the two VOCs, *cis*-3-hexenyl acetate and myrcene, reached a significant difference (F = 7.550; df = 232, *p* < 0.01).

### 2.10. Adult Mimics + Different Concentrations of VOCs and Adult Mimics Attraction Experiment

**Figure 7:**
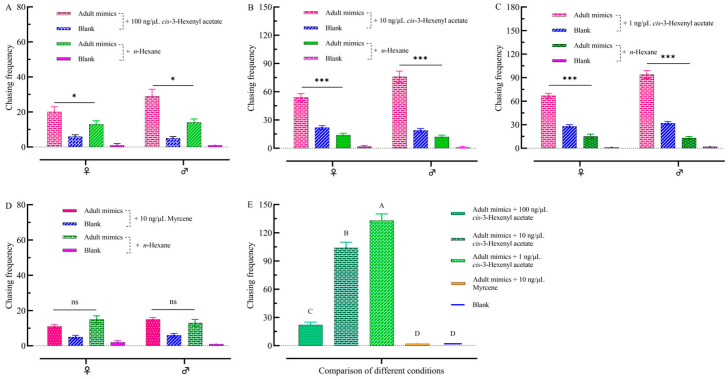
Comparison of numbers of adult visits to adult mimics + VOCs. (**A**) Comparison of the numbers of adult chases to adult mimics + 100 ng/μL *cis*-3-hexenyl acetate with blank + *n*-hexane. (**B**) Comparison of the numbers of adult chases to adult mimics + 10 ng/μL *cis*-3-hexenyl acetate with blank + *n*-hexane. (**C**) Comparison of the numbers of adult chases to adult mimics + 1 ng/μL *cis*-3-hexenyl acetate with blank + *n*-hexane. (**D**) Comparison of the numbers of adult chases to adult mimics + 10 ng/μL myrcene with blank + *n*-hexane. (**E**) Comparison of the numbers of adult chases to adult mimics with blank. Histograms are expressed as mean ± SD. One-way ANOVA was used to determine differences in the numbers of adult chases (*p* < 0.05). Post hoc test is used by LSD. * indicates significant differences in the frequency of adult chases. ***: *p* < 0.001. ns indicates no significant differences in the frequency of adult chases. Capital letters indicate significant differences in adult visits in five different conditions (Figure 7E).

The data showed that there was a difference in the frequency of chases by male and female adults to the different conditions (Figure 7). The total number of chases by adult FAWs to the 100 ng/μL *cis*-3-hexenyl acetate + adult mimics was 39, for males 20 (51.28%) and females 19 (48.72%), which was significantly greater than that to the adult mimics + *n*-hexane (F = 2.991; df = 4, *p* < 0.05) (Figure 7A). The total number of chases by adult FAWs to the 10 ng/μL *cis*-3-hexenyl acetate + adult mimics was 130, for males 54 (41.53%) and females 76 (58.46%), which was significantly greater than that to the adult mimics + *n*-hexane (F = 1.734; df = 4, *p* < 0.01) (Figure 7B). The total number of chases by adult FAWs to the 1 ng/μL *cis*-3-hexenyl acetate + adult mimics was 161, for males 94 (58.39%) and females 67 (41.61%), which was significantly greater than that to the adult mimics + *n*-hexane (F = 0.308; df = 4, *p* < 0.01) (Figure 7C). The total number of chases by adult FAWs to the 10 ng/μL myrcene + adult mimics was 26, for males 15 (57.70%) and females 11 (42.30%), which was not significantly greater than that to the adult mimics + *n*-hexane (F = 0.160; df = 113, *p* > 0.05) (Figure 7D). Male and female adult moths showed different levels of attraction to three different concentrations of *cis*-3-hexenyl acetate (100 ng/μL, 10 ng/μL, and 1 ng/μL) + adult mimics. The total number of chases for each concentration was as follows: 22 (8.55%) for 100 ng/μL, 104 (40.15%) for 10 ng/μL, and 133 (51.35%) for 1 ng/μL. The total number of chases for 1 ng/μL *cis*-3-hexenyl acetate + adult mimics was significantly different from 100 ng/μL and 10 ng/μL (F = 350.604; df = 2, *p* < 0.01) (Figure 7E). This indicates that the 1 ng/μL concentration of *cis*-3-hexenyl acetate is particularly attractive to adults. This indicates that low concentrations of *cis*-3-hexenyl acetate play an essential role in the mating process. A concentration of 10 ng/μL myrcene barely induces mating behavior.

### 2.11. Maize Green Leaf VOC cis-3-Hexenyl Acetate Trapping Experiment

**Figure 8:**
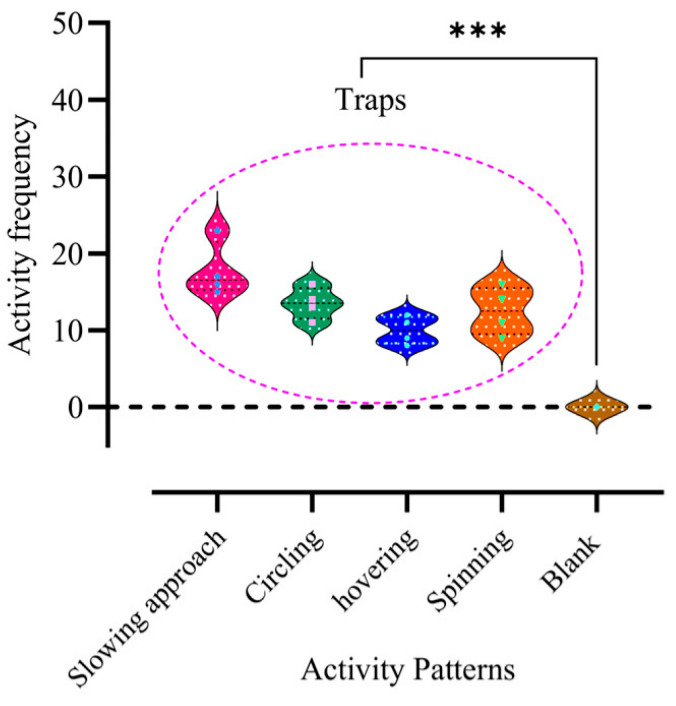
Activity frequency of male and female adults near bucket traps compared with the control. Chi-squared test was used to determine differences in the number of adult activity patterns (*p* < 0.05). ***: *p* < 0.001.

The results showed that traps infused with the VOC *cis*-3-hexenyl acetate were significantly more attractive to adult FAWs than control traps, indicating that *cis*-3-hexenyl acetate is a key VOC in the identification of maize as a suitable host plant by adult moths. In addition, this study observed that when flying near *cis*-3-hexenyl acetate bait traps, both male and female adult moths tended to slow down, circle, or hover around the traps. Peak activity for both sexes occurred around 2:00 a.m., with more than 20–30 adults congregating near *cis*-3-hexenyl acetate traps (Figure 8), and the activity of male and female adults near the traps showed a highly significant difference compared with the control (*X*^2^ = 216.00^a^, df = 4, *p* < 0.01), indicating that this VOC significantly alters the uniformity of the spatial distribution of adult moths. This suggests that *cis*-3-hexenyl acetate is critical in stimulating host recognition in adult moths.

## 3. Discussion

Finding a suitable host plant is a survival-driven task for insects that is accomplished in two stages: selection and location. Location is achieved through the combined use of olfactory and visual cues, while the selection phase involves contact with the host to confirm the acceptability of its gustatory cues [9]. Both visual and olfactory cues can influence insect preferences in host plant selection. Visual and chemical cues have been reported to play a role in host location in *Mamestra brassicae* (Lepidoptera: Noctuidae) [28] and *Diaphorina citri* (Hemiptera: Liviidae) [45]. To gain a more complete understanding of the host-finding mechanisms of FAWs, it is necessary to assess the response of FAWs to the interaction of olfactory and visual cues, known as bimodal cues. Understanding the utility of visual and olfactory maize leaf cues in host finding by adult FAWs will enable targeted approaches to insect population control.

FAW larvae typically live and feed on their host plant, maize, until they pupate, while adults adopt an aerial lifestyle, focusing primarily on finding mates and host plants and bearing the responsibility for producing offspring. The Preference Performance Hypothesis (PPH), or “mother knows best” concept, suggests that adults tend to prefer host plants that are conducive to larval survival, especially when the mobility of immature stages is less than that of adults [46,47]. The results showed that the larvae caused the most damage to maize at the small trumpet mouth stage, indicating that they prefer to feed on maize at this stage, which is more attractive to larvae and more suitable as a feeding and oviposition host plant for FAWs. Spectral data from this study show that the spectral peak (490~570 nm) and spectral curve of the same maize variety are basically consistent across different growth stages, and different maize varieties have almost the same spectral peak (490~570 nm) and spectral curve at the same growth stage. This suggests that leaf color is not the key to influencing the differential selection of different growth stages of maize by adults. Subsequent single-color mimics without olfactory interference also confirmed our hypothesis, with lower visit frequencies of male and female adults to single vision, indicating that adults have some color recognition abilities. This is consistent with the results of previous studies, showing that FAWs have four opsin genes associated with positive phototaxis [48] and demonstrating the phototactic behavior of FAWs [29], which are widely monitored and managed using light traps in China. Previous studies have shown that nocturnal insects consistently have larger eyes, but not larger numbers of antennal sensilla, compared with diurnal species; this is consistent with the relative importance of olfaction versus vision for a species being highly dependent on its life history, rather than solely on whether a species is diurnal or nocturnal. This suggests that although FAWs can receive light of approximately 300~600 nm, it is unknown whether FAWs can discriminate light spectra (color) [49]. Adult males and females were more active during single-odor attraction, confirming that adults are odor sensitive. Previous work has shown that naive individuals of the diurnal species show a selective preference for food sources based primarily on visual cues, whereas the nocturnal species show a preference for olfactory cues [50]. That is, more for vision in diurnal species and more for olfaction in nocturnal species [51]. When the relative importance of visual and olfactory cues was tested, olfactory cues were more attractive to adults than visual cues, and leaf odors played a dominant role in adult host location. In the dual-choice bioassay of visual and olfactory cues to adults, the visit frequency of adults to maize plants in their natural state was significantly higher than that of visual attraction alone or olfactory attraction alone, indicating that the combination of visual and olfactory stimuli is more attractive to adults. The olfactory and visual sensory systems of moths play important roles in flower finding [50]. In addition to acting as long-range attractants and foraging cues, floral odors also act as enhancers of other sensory cues, exerting direct and indirect effects [52,53]. This is consistent with our finding of more frequent visits to maize when both visual and olfactory cues were available and suggests that FAWs, like many other nocturnal Lepidoptera, use both vision and olfaction for host plant selection.

The recognition of host plants by insects is essential to ensure their own survival and that of their offspring, as these plants are considered not only as food substrates, but also as suitable sites for oviposition and refuge [54,55]. Plant VOCs, acting as olfactory cues, play an important role in the host location process of phytophagous insects [38,56]. Lepidopteran insects rely heavily on chemical cues, such as VOCs, to find suitable host plants for oviposition [57]. In this study, VOCs were further detected and analyzed from the maize leaf, which indicated that there are differences in the concentrations and types of VOCs during the small trumpet mouth stage among three different maize varieties. VOCs that are common and have higher content can be initially screened as olfactory identification indicators, and six VOCs were selected for EAG response. The results show that there are differences in physiological responses to different substances at the same concentration, and there are also differences in EAG responses to the same concentration of different substances. This indicates that the EAG response of FAWs is related to the category and concentration of VOCs, among which the green leaf VOC *cis*-3-hexenyl acetate has the strongest EAG response. Insects typically rely on their olfactory system to detect VOCs from host plants and make decisions about oviposition [58]. This reliance on olfaction is critical for the survival and reproduction of many species, including FAWs, which use both olfactory and visual cues to locate suitable hosts. The integration of these sensory inputs is key to their foraging and reproductive behavior. Understanding these mechanisms can provide valuable insights for developing strategies to effectively manage pest populations. Based on the EAG data, *cis*-3-hexenyl acetate and myrcene were tentatively selected for further testing of volatile attraction in a Y-tube. There were differences in the attraction of male and female adults to high concentrations of *cis*-3-hexenyl acetate, and the number of adult visits increased sequentially as the concentration of *cis*-3-hexenyl acetate decreased. Studies have shown that oviposition and olfactory preference for antennal-active compounds by adult female FAWs are sensitive to the active VOC *cis*-3-hexenyl acetate at 100 μg (*p* < 0.05), 10 μg (*p* < 0.001), and 1 μg (*p* < 0.001) [8]. Analyzing the reasons, it is possible that the experimental samples in our experiments are analogs sought based on GC-MS results and are different from true plant volatiles, which can be a complex mixture of odors, and our experimental samples are likely to be closer to plant VOCs under low-concentration conditions. There are also differences in the response of male and female adults to the myrcene concentration gradient, with males more attracted to 10 ng/μL myrcene and females more attracted to 1 ng/μL myrcene. A complex interaction between VOC concentration and sensory perception mechanisms was demonstrated in combination with the results of the EAG analysis [59]. The perception and selectivity of FAWs for plant VOCs are dose dependent.

Both visual and olfactory cues increase the attractiveness of host plants [60]. The interaction between color and VOCs has been confirmed in the fruit fly, *Drosophila suzukii* Matsumura [61]. This study’s model and behavioral observations of volatiles again support the view that both visual and chemical cues play a critical role in insect host selection [61,62,63,64]. Some attractions are mediated by a single VOC [65]. The behavioral experiments in this study show that adult FAWs are strongly attracted to low concentrations of *cis*-3-hexenyl acetate. This suggests that *cis*-3-hexenyl acetate is involved as an important chemical cue in the host location of the 1 ng/μL FAW. This result may suggest a threshold effect or a specific concentration range that is more attractive to moths. The test VOCs in our experiments are analogs sought based on GC-MS results and differ from true plant VOCs, which can be a complex mixture of odors, and our experimental specimens are likely to be closer to plant volatiles under low concentration conditions. Further research is also needed to fully understand this effect. There are already experiments confirming that 1 ng/μL *cis*-3-hexenyl acetate is an important VOC that stimulates oviposition in maize plants [8]. In addition, this study found that the significant difference in the number of VOCs + mimics chases by male and female adult moths compared with the control indicates that the adults have some visual recognition ability. Although male and female moths can recognize conspecifics, their recognition ability is limited. This finding underscores the complexity of insect sensory systems and suggests that while visual cues play a role in mate recognition, other factors such as chemical signals may also be critical in the overall process of mate identification and attraction. *cis*-3-hexenyl acetate has the effect of enhancing the mating behavior of adult FAWs. *cis*-3-hexenyl acetate is a type of green leaf volatile (GLV), a compound that may have the potential to act as a sex pheromone used by FAWs to attract mates. The discovery that *cis*-3-hexenyl acetate enhances the mating behavior of FAWs opens new avenues for research and potential pest management applications. It highlights the complexity of insect–plant interactions and the potential to harness these interactions for more effective and sustainable pest control methods.

## 4. Materials and Methods

### 4.1. Experiment Location

**Figure 9:**
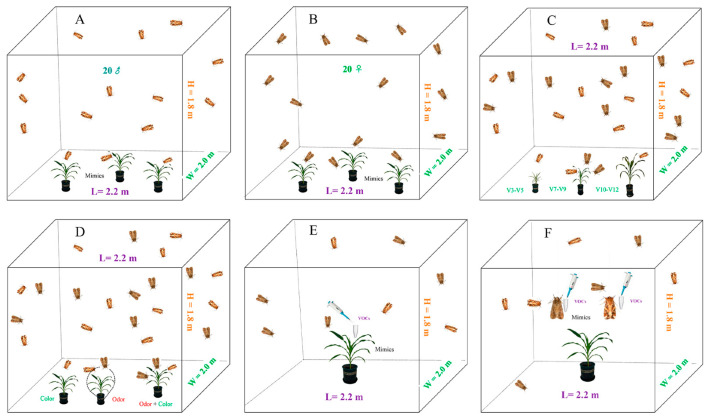
Schematic diagram of the equipment associated with the adult attraction test. (**A**) Vision test for male adult visiting single color. (**B**) Vision test for female adult visiting single color. (**C**) Adults visiting three different stages of maize plant attraction tests. (**D**) Adults visiting three different condition attraction tests. (**E**) Adult visiting maize plant mimics + VOCs tests. (**F**) Adults visiting adult mimics + VOCs attraction tests.

Indoor observation experiments were conducted at night under a 20 W red LED light in two net rooms (2.2 × 2.0 × 1.8 m) (Figure 9), and VOC *cis*-3-hexenyl acetate hood net validation was conducted in two net rooms (6 × 1.5 × 2.5 m) of the green room in Xinxiang (113°70′ E, 35°′ N), Henan Province, east-central China. The temperature and relative humidity at the experimental site were controlled to 23–28 °C and 70 ± 2%, respectively, during the experiments. The frequency of male and female adult visits to each attractant was simultaneously observed and recorded by two researchers at night.

### 4.2. Insects and Plant Material

FAWs were obtained from the Institute of Plant Protection, Chinese Academy of Agricultural Sciences. The larvae were fed with artificial food in an incubator at 27 ± 2 °C, 65 ± 10% relative humidity (RH), and a photoperiod of 16:8 L/D. All adults used in the experiments were healthy individuals after eclosion. The test adults were placed in a net the day before the start of the experiment.

Sweet maize (Green Pioneer FI), waxy maize (Zheng Bai Tian Nuo No. 2), and regular maize (Zheng Dan 958) plants (Henan Qiu Le Seed Industry Technology Co., Ltd., Zhengzhou, China) were planted in the experimental field and greenhouse of the National Biological Breeding Industry Innovation Center in Yuanyang County, Xinxiang City, Henan Province from June to August 2023 and in January 2024, with the provision of water and fertilizer (granulated compound fertilizer, total nutrients: N + P_2_O_5_ + K_2_O ≥ 42%, 8–10 grains per plant, Henan Jinyuan Seed Industry Co., Zhengzhou, China), but no pesticide treatment. Average temperatures were about 31/20 (day/night) in a greenhouse. Plants with V3–V5 (seedling stage), V7–V9 (seventh to ninth leaf collar) (small trumpet stage), and V10-V12 (tenth to twelfth leaf collar), (large trumpet stage) were used for visual-only attraction experiments.

Leaf mimics: To eliminate visual interference from volatile compounds emitted by plant leaves, a self-adhesive matte cold lamination film (purchased from Taobao) was used to seal fresh maize plant (leaf) samples in this study. A single visual recognition identification test and a VOCs + leaf mimics attractant test were conducted.

Adult mimics: To eliminate visual interference from volatile compounds emitted from the adult body, this study used a self-adhesive matte cold lamination film (purchased from Taobao, Jinhua, China) to seal adult body samples. The visual-only identification test and VOCs + adult mimic attractant test were performed.

### 4.3. Field Assessment of Larval Damage

In this study, we analyzed field larval damage on three different maize varieties (Green Pioneer FI, Zheng Bai Tian Nuo No. 2, and Zheng Dan 958) planted at different time periods (seedling stage, small trumpet stage, large trumpet stage, and heading stage) using the five-location sampling technique.

### 4.4. Spectral Reflectance

We used an ASD Fieldspec 4 spectrometer (Beijing LICA United Technology Limited, Beijing, China) to collect hyperspectral reflectance data of maize leaves under cloudless and calm weather conditions around noon, when the sun is at its zenith, with a solar zenith angle generally less than 45°. The azimuth angle between the sun and the row direction varied from 0° to 85°. The spectral resolution of the ASD Fieldspec 4 was 3 nm@700 nm, 10 nm@1400 nm/2100 nm. Representative areas within the plant plots were used for spectral measurements. Prior to measuring a maize leaf, the spectrometer was calibrated using a reflectance whiteboard. As shown in Figure 3, nine sets of reflectance spectra were collected. To ensure that the results were reliable and consistent, each maize variety was replicated fifty times.

### 4.5. Visual Responses of FAWs During Host Search

To investigate the differences in visual cues at different growth stages of the same variety, maize plants were first sealed with ultraviolet-transparent matte plastic film (Wyda^®^) to prevent the emission of a maize odor. The sealed maize plants of the same variety at different growth stages (V3–V5, V7–V9, and V10–V12) were placed together with their pots in a net room (2.2 × 2.0 × 1.8 m) with a distance of about 0.7 m between each plant. To acclimate the moths to the experimental conditions, 20 active, unmated adult males or females were placed into the net chamber through a side zipper 10 h before the start of the observation period. To avoid positional effects, the position of the plants was rotated clockwise every 3 h.

Between 10 p.m. and 7 a.m., we calculated the total number of adults attracted by each treatment. Each adult that flew toward the leaves was considered a visit, whether it simply approached the leaves (about 5 cm or less) or landed on the leaves or the plastic bags. All experiments were conducted at night under a red LED light (20 W) to minimize visual cues.

All indoor behavioral observation experiments were repeated three to four times to maintain the reliability and integrity of results, and all observation experiments were primarily manual, supplemented by video. Males and females were observed separately in all experiments. The frequency of visits by male and female adults was simultaneously observed and recorded by two researchers under red light.

Each time an individual stopped on a plant (for at least 3–5 s), extended its proboscis, touched the plant with its body, and flew away from the plant, we considered it a visit. If an individual flew away from a plant to a distance of less than 0.5 m and returned to the same plant, we still considered it a visit (the same criteria were used for subsequent observations).

### 4.6. Olfactory Responses of FAWs During Host Search

To investigate the effect of olfactory cues alone on host identification, we first covered maize plants with black plastic bags with small holes to eliminate visual cues. The bags were perforated with tiny holes (about 1 mm in diameter) to allow the odor from the leaves to escape to the outside environment. The adults could easily detect the typical strong odor of the maize leaves. The maize plants and their pots were then placed in a net (2.2 × 2.0 × 1.8 m) with about 0.7 m between each plant. Ten hours before the start of the observation period, twenty active, unmated adult male or female moths were placed separately in the two nets through a side zipper. To avoid positional effects, the position of the plants was rotated clockwise every 3 h. All experiments were performed at night under red LED light (20 W) to avoid visual cues. The observation period was from 22:00 p.m. to 07:00 a.m. Three different maize varieties were observed in three replications for each variety. The frequency of visits by male and female adults was simultaneously observed and recorded by two researchers under red light.

### 4.7. Visual and Olfactory Responses of FAWs During Host Search

Six maize plants in their natural growth state at the small trumpet stage (with approximately 0.7 m between each plant) were separately placed in the two net rooms (2.2 × 2.0 × 1.8 m) through a side zipper to observe the frequency of adult male or female visits to the host plant. Twenty active, unmated adult males and females were separately introduced into the two net rooms through a side zipper 10 h prior to the start of the experiment. The observation period was from 22:00 p.m. to 07:00 a.m. Visiting activity of adult males and females was primarily observed manually, supplemented by video recording under LED light (20 W). The experiment was replicated three times. The frequency of visits by adult males and females in the three different conditions was simultaneously observed and recorded by two researchers under red light.

### 4.8. Collection and Analysis of Maize Leaf VOCs

Combined with the results of the field study, the larvae preferred to feed on small trumpet stage maize, and volatile extraction was performed on small trumpet stage maize in this study. A dynamic headspace sampling device was used to collect VOCs [66] with a slight modification. Fresh maize without pest damage was placed in a 41 × 45 cm^2^ plastic oven bag (Reynolds, Richmond, VA, USA) for headspace extraction. To prevent gas leakage during sampling, industrial twist ties were used instead of traditional hemp twine to seal the bags. An airflow (500 mL/min) generated by a vacuum pump (QC-1S; Beijing Municipal Institute of Labor Protection, Beijing, China) was purified with activated charcoal (10–24 mesh) and pumped into the bag. The air stream was then passed through the column for 6 h. Prior to the collection of volatiles, the absorbent columns (a glass filter, ID: 3 mm; containing 100 mg Porapak-Q 80–100 mesh; Supelco, Bellefonte, PA, USA) in the experiments were cleaned sequentially with 1 mL ethyl ether and 2 mL dichloromethane and then dried in a fume hood. VOCs adsorbed on the Porapak-Q column were eluted with 5 µL ethyl caprate and 500 µL dichloromethane. The extract was concentrated to 100 μL with a nitrogen-blowing instrument and stored at −20 °C for further use in GC-MS analyses. This sampling procedure was repeated four times for each sample. VOCs were collected from 10:00 p.m. to 4:00 a.m. the following day. Then, 1 µL of the samples was injected into a gas chromatography–mass spectrometry (GC-MS) system (Agilent7890B-7000D, Beijing, China, https://www.agilent.com/) with a DB-5 column (30 cm × 0.25 mm × 0.25 μm, Agilent, Beijing, China, https://www.agilent.com/) for VOC analysis. Nitrogen (2.0 mL/min) was used as the carrier gas. The oven temperature was maintained at an initial 40 °C for 5 min, programmed at 3 °C/min to 80 °C, then 15 °C/min to 220 °C and held for 3 min. Compound identifications were first obtained by searching the NIST11 database (Scientific Instrument Services, Inc., Ringoes, NJ, USA), and then the retention times and mass spectra of the candidate compounds were compared with the synthesized standards to determine the reliability of the identifications.

### 4.9. Electrophysiological (EAG) Experiment

This study began with a preliminary screening for VOCs using the EAG device. We initially screened six VOCs for EAG analysis in this study. After cutting approximately 2 mm from the tips of adult fall armyworm antennae, the antennae were cut from the base and attached to electrodes with conductive adhesive for EAG measurements (Syntech, Germany, model PRG-2). Diluted VOCs were applied evenly in 30 μL aliquots to filter-paper strips (4 × 0.5 cm), and the filter-paper strips were placed in Pasteur pipettes. The ends of the pipettes were connected to a stimulus gas control device, with each stimulation lasting 0.1 s and a 30 s interval between two consecutive stimulations. The antennae of each adult were replicated six times for the same sample, with only one antenna used per adult moth. The sequence of compound tests was control (*n*-hexane), VOCs, and control again. The EAG values (R) of the VOCs were compared with the average EAG values (C1, C2) of the two reference compounds before and after to obtain the relative EAG values (V), calculated as V = 2R / (C1 + C2).

### 4.10. Y-Tube Olfactometer Experiment

Based on the results of the EAG experiments, this study further screened the VOCs using a Y-tube olfactometer. Y-tube experiments were conducted at 25 ± 2 °C and 50–60% RH. Each adult was dropped individually into the base of the Y-tube, placed horizontally on the table, and could choose either arm. The maximum duration of each trial was 10 min. For each trial, a choice was recorded if the insect entered an arm 5 cm from the junction and remained motionless for more than 20 s. Those that did not make a choice were also recorded. The positions of the odor source and control were reversed, and the walls of the tubes were cleaned with water after every 5 adults were tested. During detection, 20 μL of the test standard was spotted uniformly onto a folded filter-paper strip (0.4 cm × 0.4 cm), which was then placed in one end of an odor source bottle as the treatment group. The other end of the odor source bottle contained a filter-paper strip spotted with an equal volume of *n*-hexane as the control.

### 4.11. Test VOCs and Mimics

The VOCs *cis*-3-hexenyl acetate, 2-ethylhexyl acetate, myrcene, linalool, *n*-tridecane, and *cis*-anethol were purchased from Aladdin Reagent Company (Shanghai, China) (https://www.aladdin-e.com/customersupport, accessed on 18 March 2023) and diluted in *n*-hexane (>98% purity, CAS 110-54-3) in appropriate proportions to prepare for (electrophysiology) EAG, Y-tube, and mimics + VOCs attraction tests.

Maize plants and adult mimics were sealed with matte plastic sealing film (Wyda^®^) to prevent odor emissions. During the observation process, the mimics were placed in a net the day before the experiment began.

### 4.12. Mimics (Leaf or Adult Sealed Model) + Different Concentration VOC Attraction Experiment

Based on the results of the first experiment, the following two reagents were selected: *cis*-3-hexenyl acetate (CAS: 3681-71-8) and myrcene (CAS: 123-35-3). The experiment was divided into eight groups: 100 ng/μL *cis*-3-hexenyl acetate + maize leaf mimics, 10 ng/μL *cis*-3-hexenyl acetate + maize leaf mimics, 1 ng/μL *cis*-3-hexenyl acetate + maize leaf mimics, 10 ng/μL myrcene + maize leaf mimics, 100 ng/μL *cis*-3-hexenyl acetate + adult mimics, 10 ng/μL *cis*-3-hexenyl acetate + adult mimics, 1 ng/μL *cis*-3-hexenyl acetate + adult mimics, and 10 ng/μL myrcene + adult mimics. Each group was observed separately.

In this study, a 250 μL centrifuge tube was attached to the sealed maize leaf mimics. A pipette was used to add 200 μL of the VOCs to be tested and *n*-hexane to the 250 μL centrifuge tube. Ten hours before the start of the observation period, twenty active, unmated adult males and females were placed in the two net rooms through a side zipper opening. The visits of adult males and females to the mimics were recorded. Observations were made from 10 p.m. to 7 a.m. the following day. All experiments were performed at night under red LED light (20 W) to avoid visual cues.

Six adult male and female mimics and three blank models were suspended in a net room 1.6 m above the ground. A pipette was used to add 200 μL of the volatiles to be tested and *n*-hexane into the 250 μL centrifuge tube of the mimics. Every three hours, the position of the mimics was randomly changed, and 200 μL of VOC was replenished with a pipette. Ten hours before the start of the observation period, twenty active, unmated adult males and females were placed separately in the two net rooms through a side zipper opening. The visits of adult males and females to the mimics were recorded. Observations were made from 10 p.m. to 7 a.m. the following day. All experiments were performed at night under red LED light (20 W) to avoid visual cues. The frequency of adult male and female visits to each attractant was simultaneously observed and recorded by two researchers under red light.

### 4.13. Maize Green Leaf VOC cis-3-Hexenyl Acetate Hood Net Validation

This experiment was designed to evaluate the efficacy of *cis*-3-hexenyl acetate as an attractant for adult moths under controlled conditions that mimic the natural environment. The *cis*-3-hexenyl acetate attraction experiment was conducted in two net rooms, each measuring 6 m long, 1.5 m wide, and 2.5 m high. The day before the experiment, 80 female and 80 male adult moths were introduced through a side zipper in the long side at the middle position of each net room. Cotton pellets impregnated with 200 μL *cis*-3-hexenyl acetate bucket-type traps (Beijing Zhongjie Sifang Biotechnology Co., Ltd., Beijing, China) containing VOCs were placed at one end of the net room and a control (*n*-hexane) at the other end of the chamber and suspended 20 cm above the top of V12–V16 (twelfth to sixteenth leaf collar) maize plants, which were approximately 1.6 m high. The activity characteristics of the adult moths in response to the lures were observed and recorded. The experiment was repeated five times to ensure the reliability of the observations. Each day, the number of active moths was counted, and dead moths were removed from the net and replaced with healthy, live adults. The position of the traps was changed every 3 h. The frequency of male and female adult visits to each attractant was simultaneously observed and recorded by two researchers under red light.

### 4.14. Data Analysis

Data statistics from the dose–response, EAG, and adult and larval behavior experiments were analyzed using SPSS Statistics 27.0 (IBM Corp, New York, NY, USA). GraphPad PRISM 10.0 software (GraphPad Software, La Jolla, CA, USA) was used to generate graphs. Two-sample analysis was performed using Welch’s *t*-test (a = 0.05). A chi-squared test (L-R *X*^2^ degrees of freedom) was used to detect significant differences between leaf-only and leaf-cover reflectance spectra. The least significant difference test was used to compare EAG and Y-tube responses in adults. One-way ANOVA was used to detect significant differences between males and females during host exploration. Levene’s test was used for homogeneous variances and least significant difference (LSD) for multiple comparisons with homogeneous variances. The nonparametric rank sum test was used for nonhomogeneous variances.

## 5. Conclusions

In conclusion, the results suggest that 1 ng/μL *cis*-3-hexenyl acetate may serve as an attractive lure for *S. frugiperda*. This compound, identified by GC-MS analysis and validated in EAG, Y-tube, mimics, and trap assays, was shown to act as a primary olfactory cue, improving host recognition efficiency and stimulating certain (initial) acts of courtship behavior. In this study, host location experiments showed that while visual and olfactory stimuli together resulted in the strongest attraction, olfactory stimuli alone attracted more individuals than visual stimuli alone, indicating that visual cues play a secondary role in host location for FAWs.

## Figures and Tables

**Table 1:** Volatile organic chemicals from the V3–V5 (third to fifth leaf collar) stage of leaves of 3 maize varieties.

VOCs	CAS	Sweet Maize	Common Maize	Glutinous Maize
*p*-xylene	106-42-3	7.05 ± 0.51	23.82 ± 2.58	-
4-Ethylacetophenone	937-30-4	-	12.95 ± 0.89	1.28 ± 0.09
*cis*-Anethol	104-46-1	7.40 ± 0.32	5.33 ± 0.31	8.56 ± 0.83
*n*-Tridecane	629-50-5	3.23 ± 0.03	4.43 ± 0.14	4.37 ± 0.54
Benzaldehyde	100-52-7	3.76 ± 0.21	5.18 ± 0.45	3.23 ± 0.31
Nonanal	124-19-6	8.47 ± 0.54	-	28.24 ± 2.56
2-Ethyl-1-hexanol	68526-83-0	-	5.16 ± 0.03	3.24 ± 0.01
*cis*-3-Hexenyl acetate	3681-71-8	66.37 ± 3.89	98.37 ± 3.57	50.21 ± 3.31
Phenethyl acetate	103-45-7	6.85 ± 0.51	16.42 ± 0.94	-
2-Ethylhexyl acetate	103-09-3	6.53 ± 0.37	51.03 ± 2.11	4.43 ± 0.09
Perillen	539-52-6	12.90 ± 1.02	-	-
*trans*-Caryophyllene	87-44-5	4.28 ± 0.11	-	13.41 ± 1.21
Myrcene	123-35-3	15.96 ± 1.65	10.96 ± 1.32	9.44 ± 0.87
Linalool	78-70-6	3.97 ± 0.02	5.37 ± 0.34	3.60 ± 0.15
Octene	111-66-0	7.55 ± 0.27	-	-

## Data Availability

The original contributions presented in the study are included in the article, further inquiries can be directed to the corresponding author.
